# Femoral nonunion with segmental bone defect treated by distraction osteogenesis with monolateral external fixation

**DOI:** 10.1186/s13018-017-0684-y

**Published:** 2017-11-25

**Authors:** Qun Zhang, Wei Zhang, Zhuo Zhang, Licheng Zhang, Hua Chen, Ming Hao, Junhao Deng, Peifu Tang

**Affiliations:** Department of Orthopaedics, Chinese PLA General Hospital, No. 28 Fuxin Road, Beijing, 100853 People’s Republic of China

**Keywords:** Monolateral external fixation, Distraction osteogenesis, Bone defects, Femoral nonunion

## Abstract

**Background:**

Currently, the common treatment for femoral nonunion with large segmental bone defect is difficult and complex. The effective surgical methods are rare, include vascularized bone grafting, Masquelet technique and Ilizarov distraction osteogenesis. The objective of this study is to investigate the outcomes of segmental femoral defects treated with monolateral external fixation using the distraction osteogenesis.

**Methods:**

We retrospectively analyzed patients with femoral nonunion with segmental bone defects (> 6 cm) between January 2010 and January 2014 in our single trauma center. All patients were treated by distraction osteogenesis with monolateral external fixation. All surgeries were performed by the same surgeon. Bone union, duration of distraction osteogenesis in days, time to consolidation in months, external fixation index (EFI), complications, and additional surgical interventions were recorded postoperatively. The modified Application of Methods of Illizarov (ASAMI) criteria were used to evaluate the operative effectiveness.

**Results:**

Forty-one patients were enrolled in this study for analysis. The length of the bone defect ranged from 6 to 17 cm. All patients eventually achieved healing, and no patient experienced recurrence of infection or newly developed infection. The average time needed for healing was 13 months. In terms of the incidence of complications, 3 cases axial deviations, 5 cases docking site nonunion, 23 cases pin-tract infection, 14 cases knee joint stiffness or their joint mobility declined, 2 cases osteogenesis insufficient in the distraction area,1 case refracture, and 2 cases loose external fixation pins. In terms of the evaluations of fracture healing and function, 30 patients excellent, 6 patients good, 5 patients fair, and 0 patient poor. In terms of postoperative function evaluations, 21 patients excellent, 9 patients good, 7 patients fair, and 4 patients poor.

**Conclusion:**

For patients with femoral nonunion with large segmental bone defects, the monolateral external fixation can provide effective stability, improve compliance, and reduce complications.

**Electronic supplementary material:**

The online version of this article (10.1186/s13018-017-0684-y) contains supplementary material, which is available to authorized users.

## Background

The common causes of posttraumatic femoral nonunion with large segmental bone defect (> 6 cm) include acute bone loss, bone ischemia atrophy in nonunion sites, and surgical removal of dead bone and sclerotic bone after infection [1, 2].Current treatment for the disease, in addition to the need of addressing the issue of bone nonunion with bone defect, soft tissue defect, nearby joint stiffness, deformities (rotation, angulation, and shortening), infection and many other issues should also be treated simultaneously [3–5]. At present, the common treatments include vascularized bone grafts (such as ribs, ilium, and fibula), intramembranous osteogenesis technique (Masquelet technique), and Ilizarov distraction osteogenesis [6–10]. Among them, Ilizarov distraction osteogenesis can simultaneously address the issues of infection, bone and soft tissue defects, and correction of deformities and eventually achieves the fracture healing. It is one of the most effective therapeutic strategies for posttraumatic complex nonunion [5, 9, 10].Clinically, the Ilizarov circular frame, the Taylor spatial frame (TSF), the semicircular Ilizarov pin fixator, and the conventional external fixation are applied to distraction osteogenesis [11–15]. However, complications associated with external fixation systems are high. Moreover, the compliance of patients is relatively poor. Although the monolateral external fixation can provide good stability and compliance is high, there are relatively few reports that describe the outcomes of femoral nonunion treated with this external fixation system [16–18].

Therefore, we retrospectively analyzed patients with femoral nonunion with segmental bone defects who were treated with the monolateral external fixator between January 2010 and January 2014 to evaluate the effectiveness, stability, and complications of the monolateral external fixator.

## Methods

The inclusion criteria were as follows: (1) posttraumatic femoral nonunion, (2) segmental bone defect > 6 cm preoperatively and/or intraoperatively, and (3) Ilizarov technique with the monolateral external fixator. Patients who met the above criteria were included in this study. The exclusion criteria were as follows: (1) nonunion caused by primary or secondary tumor, congenital bone disease, metabolic bone disease, or severe vascular origin disease; (2) nonunion for which physiotherapy or drug therapy was used during the treatment to promote fracture healing; (3) nonunion that was combined with severe systemic organ failure; and (4) nonunion that was combined with a mental disorder.

### Surgical procedure

**Fig. 1:**
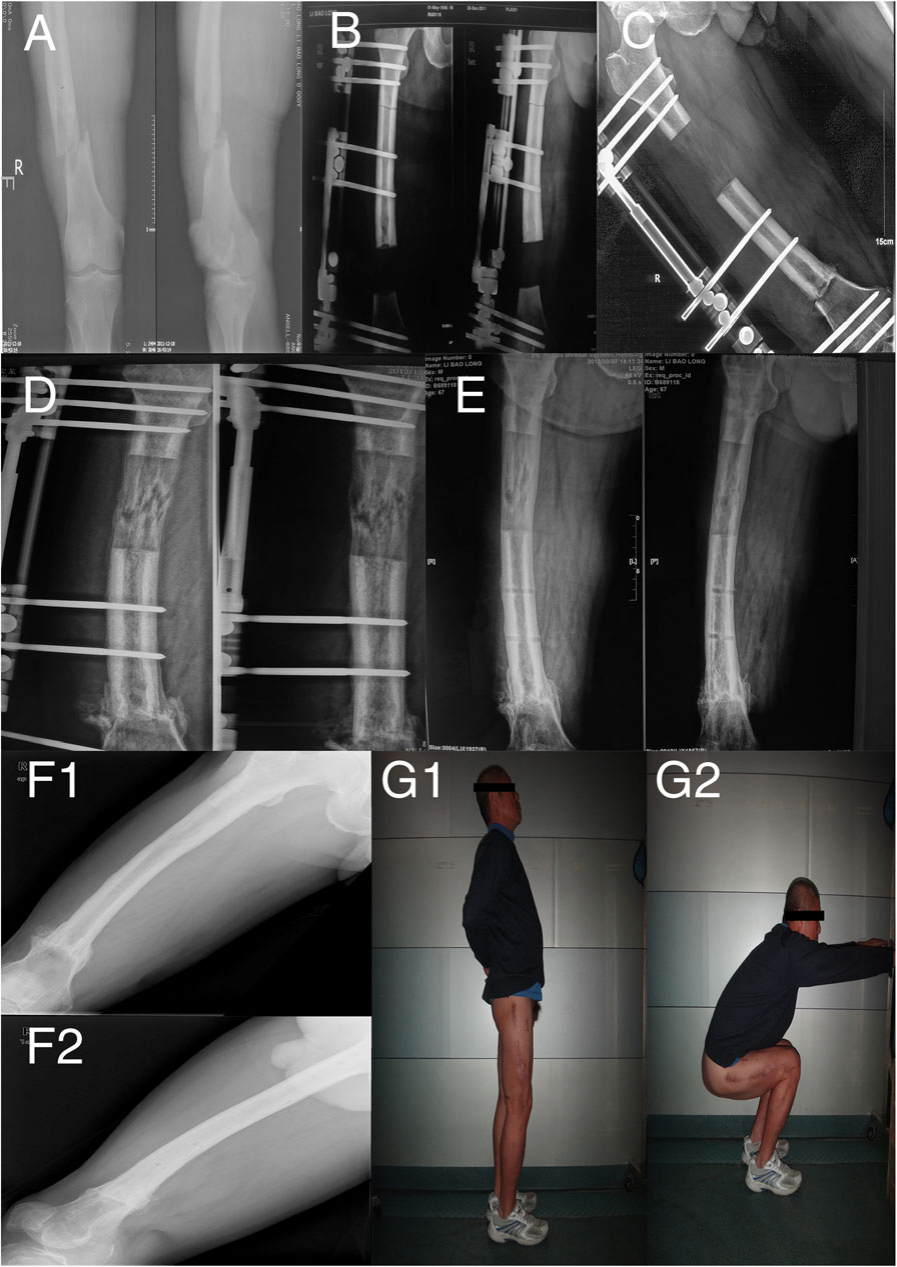
Sixty-year-old male, suffered a 2-year-long postoperative infection after fracture of left femoral shaft. **a** The X-ray showed refractures occurred 2 years later; **b** the patient was given the debridement of lesions, single-arm external fixator, and bone transport. The postoperative presentation of the X-ray demonstrated a 10-cm-long bone defect of femur. **c** The 5-month-later presentation of the X-ray demonstrated the femur length became normal. **d** The 1-year-later presentation of the X-ray demonstrated the bone grew well in the region of distraction osteogenesis and the docking site healed well. **e** The 18-month-later presentation of the X-ray with external fixator removed. **f** The 4-year-later X-ray presentation showed no infection recurred. **g** The patient showed a good function of flexion and extension of knee joint

After successful anesthesia, for patients who originally had fixation objects, the internal and external fixation objects were firstly removed to expose the nonunion site (Fig. 1a-g2). Then, dead bone, sclerotic bone, fibrous scar tissue, and infected tissue were completely debrided, and a pendulum saw was used to clean up the nonunion site until fresh bleeding healthy bone tissue was reached. By now, the length of bone defect was measured. On the basis of radical debridement, the placement of external fixation pins was initiated. The hydroxyapatite-coated external fixation pins and the extendable monolateral external fixation frame were from Orthofix, Italy. Under the image intensifier, 8–9 parallel pins were inserted at the lateral side of the femur, perpendicular to its long axis. Three pins were fixed at the proximal and distal ends of the femur, respectively, and 2–3 pins were inserted on the transported bone segment. Make sure all pins are on the same coronal plane. The external extendable monolateral fixation frame was then installed. After the osteotomy plane was determined, small-incision low-energy subperiosteal osteotomy was performed, and compression was applied at the osteotomy site. Finally, the incision was closed. If the soft tissue could not be closed during the primary phase or infection was severe, a vacuum-assisted closure device was used to cover the wound, and the wound would be closed during the second phase. The suspected tissue was taken from multiple sites during the procedure and was sent for bacteriological culture to guide postoperative antibiotic use. The surgeries were completed by the same experienced senior surgeon.

### Postoperative management

**Fig. 2:**
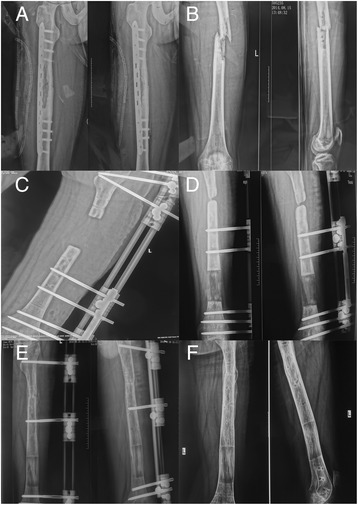
Twenty six-year-old male, suffered a 14-month-long infection after the operation using bone plates of fracture of left femoral shaft. **a** The X-ray presentation after the open debridement combined with irrigation outside the hospital. **b** After removing the internal fixator due to the runaway infection, the postoperative presentation of the X-ray demonstrated the nonunion of fracture and evident displacement, and some sequestra with bone defects could be seen locally. **c** The 1-week-later X-ray presentation after bone transport: single-arm external fixator served well, and there existed a 6-cm-long bone defect after thorough removal of sequestra and infectious tissues. **d** The 2-month-later X-ray presentation after bone transport: bone growth could be seen in the region of distraction osteogenesis, but both sides of docking site were significantly hardened. **e** The 14-month-later X-ray presentation after bone transport: the docking site healed well after debridement, autogenous bone graft, and compression. The bone grew well in the region of distraction osteogenesis and external fixation pins were partly removed. **f**: The 20-month-later X-ray presentation after bone transport: no infection recurred

For aseptic nonunion, patients were treated with broad-spectrum antibiotics for 3 days. For septic nonunion, anti-infection treatment was administered for 14 days according to the drug sensitivity results (Fig. 2a-f). Disinfection care for the pin tract was conducted every day to prevent infection. Seven to 10 days postoperatively, bone transport was initiated, with the extension based on 0.25 mm four times per day. Three days postoperatively, the patients began partial weight-bearing activities with crutches. X-rays were taken and reviewed every 2 weeks to observe the growth condition of the distraction area and whether there was axial deviation of the transported bone segment. X-ray monitoring was stopped when the limb length was achieved or when the docking site made contact. Before removing the external fixator, the compression or distraction force was gradually eliminated to ensure that the frame connection was neutral so that there was no tension in any direction. The removal of the external fixation was based on the following findings: osteogenesis is sufficient in the distraction area, fracture healing was reliable, and no deformations were found at the nonunion site and distraction area when the patient walked on full weight-bearing activities.

All relevant complications were recorded, and the corresponding treatments were clarified. Postoperative pin-tract infection was classified according to Marsh’s description [19]. The assessment of clinical efficacy was conducted by the modified Application of Methods of Illizarov (ASAMI) criteria [20].

## Results

According to the inclusion and exclusion criteria, our study enrolled 43 patients (Additional file 1). Two patients were lost to follow-up, and 41 patients were eventually included for analysis. The patients ranged in age from 26 to 76 years old, with an average age of 44 years old. There were 31 males and 10 females. Twenty-eight patients had previous open fractures, and 13 patients had previous closed fractures. The duration from the time of injury to the present ranged from 10 to 60 months, with an average of 23.4 months. The patients had received 1–9 previous surgeries: 7 patients had initial internal fixations, 21 patients had intramedullary (IM) nailing, and 13 patients had external fixation. The length of the bone defect ranged from 6 to 17 cm, with an average length of 10.1 cm. There were 33 cases of septic nonunion (21 draining and 12 quiescent nonunion) and 8 cases of aseptic nonunion. Fifteen injured limbs had combinations of rotation deformities, and 11 cases had angulation deformities. Eight patients had knee joint dysfunction. The demographic characteristics of the patients studied can be seen in Table 1.

**Table 1:** Demographic characteristics of the patients studied

Variable	Number
Total number	41
Age (years)	26–76
Gender	
Male	31
Female	10
Time since injury (months)	10–60
Number of surgeries	1–9
Patterns of initial fractures	
Open	28
Closed	13
Patterns of initial surgeries	
Plating	7
IM nail	21
External fixation	13
Patterns of bone nonunion	
Infection	33
Aseptic	8
Length of bone loss (cm)	6–17
Other types of deformities	
Rotation	15
Angular	11

The postoperative follow-up time ranged from 20 to 60 months (average 35 months). All fractures eventually achieved healing, and no patient experienced recurrence of infection or newly developed infection. The duration of distraction osteogenesis (DOG) was 60–191 days (average 110 days). The time needed for healing was 6–20 months (average 13 months). The external fixation index (EFI) was 1.15–1.52 months/cm (average 1.30 months/cm). In terms of complications, 3 patients had axial deviations, which were corrected by surgical adjustment and enhanced fixation; 5 patients had docking site nonunion, of whom 3 patients were given autologous cancellous bone grafting combined with continuous compression at the docking site to achieve healing, and 2 patients underwent the “accordion technique” to achieve healing. There were 23 cases of pin-tract infection, of which 15 cases were type A, 7 cases were type B, and 1 case was type C; 10 of those cases mainly occurred at the greater trochanter site, while the pins of 3 patients were removed and replaced, after which control was gained over the infection. Fourteen patients had knee joint stiffness and a range of knee motion declined; according to the differences in the patients’ living requirement, joint arthrolysis was performed later for 10 patients. Two patients had osteogenesis insufficient in the distraction area; 1 patient was treated with autologous cancellous bone grafting, while the other patient was treated with the “accordion technique.” One patient experienced refracture when he accidentally fell down 1 year after the external fixation frame was removed; the refracture occurred at the docking site. Since this patient previously had an infectious bone nonunion, an external fixation frame was used for fixation again, and refracture healing was achieved with the “accordion technique.” Two patients had loose external fixation pins, which were replaced with new fixation pins (see Table 2). In terms of the evaluations of fracture healing and function, 30 patients excellent, 6 patients good, and 5 patients fair. In terms of postoperative function evaluations, 21 patients excellent, 9 patients good, 7 patients fair, and 4 patients poor (see Table 3).

**Table 2:** Details of the outcomes and complications

Variable	
Follow-up in months	35(20–60)
Duration of DOG in days	110(60–191)
Time to consolidation in months	13(6–20)
EFI (months/cm)	1.30(1.15–1.52)
The number of union	41
The number of complications	
Pin-track infection	23
Wire/pin loosening	2
Reinfection in fraction site	0
Vascular/nerve injury	0
Axial deviation	3
Docking site nonunion	5
Refraction	1
Osteogenesis insufficient in distraction area	2
Knee joint rigidity	14
The number of additional surgical interventions	
Bone grafting	4
Knee arthrolysis	10
Accordion technique	3
External fixator adjustment	3
Remove/change external fixation pin	2
Fixation in refraction	1

*DOG* distraction osteogenesis, *EFI* external fixation index

**Table 3:** Evaluation of the bone and functional results

Grades	Bone results^a^	Functional results^b^
Excellent	30	21
Good	6	9
Fair	5	7
Poor	0	4

^a^Excellent result was defined as union, no infection, deformity of 7° and limb length discrepancy (LLD) of 2.5 cm; good was defined as union, with any two of the other three criteria; fair result was defined as union, with one of the other three criteria; and poor result was defined as nonunion

^b^Excellent result was defined as active, without the other four criteria; good was defined as active, with 1–2 of the other four criteria; fair was defined as active, with 3–4 of the other four criteria; and poor was defined as inactive

## Discussion

The incidence of femoral nonunion is increasing, with a recent report published in JAMA indicating that it is as high as 13.9% [21]. This increase may be related to the increasing number of patients with severe fractures (higher degrees of open and comminuted fracture) caused by high-energy injuries (traffic, high-level fall, and crush injuries). When this condition is combined with large bone segment defects (> 6 cm) and infection, it becomes even more difficult to solve [1]. Traditional treatment requires multiple operations at different stages. Only under the premise that a thorough debridement is conducted to control the infection or there is clearly no infection can the next step in the treatment strategy for bone defect repair be determined. Traditional surgical methods often cannot effectively and simultaneously solve a series of problems including bone and soft tissue defects, lower limb deformity (rotation, angulation, and shortening), fracture nonunion, and infection.

In terms of femoral nonunion with segmental bone defects, the frequently used treatment methods include vascular pedicle autologous bone grafting (such as ribs, ilium, and fibula), intramembranous osteogenesis technique (Masquelet technique), and Ilizarov distraction osteogenesis. All of these methods have their advantages and limitations. Autologous bone grafting with a vascular pedicle requires high level of microsurgical techniques; the bone supply is limited, and it will cause a secondary damage to the donor site; failure of revascularization of the transplanted bone segment will lead to the failure of fracture healing, and insufficient femoralization of the transplanted bone segment will result in poor bone strength, which then becomes prone to refracture [6, 7, 22].

Compared with the above traditional surgical method, the Masquelet and Ilizarov techniques are the main surgical methods for the treatment of large segmental bone defects of the femur [9]. With the internal fixation, the Masquelet technique allows the patients to avoid carrying a bulky circular external fixator and its associated complications, thereby increasing patient’s compliance. However, this technique has a higher requirement for the integrity of muscle soft tissue; it requires multiple operations (at least 2) and a large amount of autologous bone; it has a higher risk of re-infection and failure of revascularization and ossification of the transplanted bone region; and it is poor at correcting severe deformities [8, 9, 23]. Therefore, its surgical indications should be selected cautiously and strictly. The Ilizarov technology has unique advantages in the treatment of femoral nonunion, especially with large-segment bone defect, as it can simultaneously address infection, bone and soft tissue defects, and corrections of deformities at the primary stage. It is suitable for various types of nonunion with a lower requirement for soft tissue covering and a higher fracture healing rate [9–12, 16–18]. Our study achieved a 100% fracture healing rate, and the functional rates of good/excellent were achieved by 73.2% of patients.

The common external fixation systems with the Ilizarov technique include the Ilizarov circular frame, the TSF, the semicircular Ilizarov pin fixator, and the conventional external fixator. However, they are often full-circular or hybrid external fixation frames, which are bulky for patients to carry and affect the exercise of adjacent joints; they also have high demands in terms of the surgeon’s technique. Therefore, Harshwal used monolateral external fixation frames to treat 7 cases of femoral nonunion, and 5 cases achieved fracture healing with good function [16]. R. Rohilla compared monolateral external fixation with circular external fixation for the treatment of tibial nonunion and found that although the circular external frame could provide better stability with less screw path infection, there were no significant statistical differences in terms of fracture healing rate and functions in the two groups [24].

On the basis of our own study, we found that the monolateral external fixator has the following advantages: (1) the surgical procedure is simpler, and it is easy to promote to lower-level medical trauma centers; (2) patients have a better tolerance, and the functional exercises are more convenient, which can facilitate improved knee joint function so that the patient can return to family and society earlier; (3) it is more suitable for patients with senile or disuse osteoporosis, and the hydroxyapatite-coated external fixation pins have stronger holding power so that the risks of loosening and failure of cutting are probably lower; and (4) since the external fixation pins are fixed from the lateral side of the femur, the risk of neurovascular damage may be lower than that associated with the circular external frame. The drawback is that, compared with the circular external frame, when the soft tissue coverage of the healthy active bone tissue at the proximal and distal femur is insufficient or when the affected limb has deformities in all three-dimensional planes, the monolateral external fixation frame cannot be used. All of the 41 patients in our study group used the monolateral external fixation frame and could perform out-of-bed functional exercises early to achieve fracture healing; furthermore, the incidence of complications was lower.

It has been reported that when distraction osteogenesis was used for the treatment of femoral nonunion, the incidence of surgical complications was very high [12, 18, 25, 26], with the mean complications per patient ranging from 1.33 (20/15) to 3.55 (71/20) [18]. Our study result was 1.22 (50/41), and the average length (10.1 cm) of bone defects of the included patients was much larger than the lengths in previously reported studies (6–8.3 cm). Under a much longer treatment cycle and with more complex conditions, the incidence of complications of this study was even lower. The most common complication was still pin-tract infection, though the incidence (56.1%) of pin-tract infection in our study was also lower than those described in other reports (63–100%) [25]. This difference may be associated with better patient education, more stable fixation by external fixation pins, and less interference with soft tissue (the use of the monolateral external fixation avoids contralateral soft tissue piercing). The second common complication was knee joint stiffness. In our study, 8 patients had preoperative joint stiffness, and only 6 patients had newly developed postoperative joint stiffness or functional decline. This low incidence was probably because there was no obstruction by the external frame behind the knee joint with the monolateral external fixation, which allows the patients to be able to perform early postoperative full-range joint exercise. However, for patients with preoperative joint stiffness, circular or hybrid external fixation also have unique advantages. The surgeon can simultaneously place a trans-articular external fixation to perform traction treatment on the stiff joint, which is a defect of the monolateral external fixation. The surgeon can choose a circular external fixation to perform simultaneous correction of joint stiffness at the primary stage according to the condition of the patient’s joint function and the surgical requirement, or the surgeon can perform joint release or joint traction at the second stage after fracture healing.

In addition, the incidence of other complications is relatively lower. However, we should pay more attention to docking site nonunion and insufficient osteogenesis in the distraction area, especially in longer bone defect and poorer soft tissue condition and elderly patients with poorer osteogenic capacity. The “accordion technique” is an effective method. By giving repeated compression-distraction stimulation at the docking site or distraction area, it can induce intramembranous and endochondral osteogenesis, thereby promoting fracture healing [27]. When necessary, autologous iliac bone graft can also be a surgical option, but this procedure is often associated with damage to the donor area and insufficient bone supply. When the lengthening bone segment experiences axial deviation, an external fixation should be adjusted immediately. By increasing the contact area of the docking site and correcting poor alignment of the affected limb, the incidence of docking site nonunion can be reduced. If necessary, a small incision can be made to clean up any fibrous scar tissue at the docking site, and a limited decortication can be performed to improve the rate of fracture healing [5, 28].

This study also has some limitations. First, this study is a retrospective small-sample single-center study with a low level of evidence. Secondly, since there is no control group, we can only evaluate the advantages and shortcomings of the monolateral external fixation, which cannot prove that it is superior to circular external fixation. All of these observations require further confirmation in large-sample multi-center prospective randomized controlled trials.

## Conclusion

Compared with the traditional circular or hybrid external fixation, even for patients with large segmental femoral defects, the monolateral external fixation can provide effective mechanical stability, make better compliance for patients, and reduce operation-associated complications. Despite the associated surgical complications being still high, correct understanding and reasonable treatment strategies can minimize the pain experienced by patients and improve the surgical success rate.

## Additional file

Additional file 1: This is the raw data.(XLSX 14 kb)

## Abbreviations

ASAMI: Association for the Study and Application of the Method of Ilizarov
DOG: Distraction osteogenesis
EFI: External fixation index
IM: Intra-medullary
JAMA: The Journal of the American Medical Association
LLD: Limb length discrepancy
TSF: Taylor spatial frame
